# Emphysematous Pyelonephritis With Scrotal Extension: An Unusual Case Presentation

**DOI:** 10.7759/cureus.45065

**Published:** 2023-09-11

**Authors:** Udham Singh, Satya Narayan Sankhwar, Manoj Kumar

**Affiliations:** 1 Urology, King George's Medical University, Lucknow, IND

**Keywords:** necrotizing infection, abscess, percutaneous nephrostomy, emphysematous pyelonephritis, diabetes mellitus

## Abstract

Emphysematous pyelonephritis (EPN) is a suppurative necrotizing form of renal infection with abscess and gas formation in the renal parenchyma and perirenal tissue. EPN with scrotal extension is rare; if not recognized and treated promptly, the clinical course can be severe and life-threatening. The most common causative organism is *Escherichia coli* and association with diabetes mellitus has been found in almost all cases. Prompt control of blood sugar and intravenous antibiotics are essential steps in management. Here, we report a rare form of extensive EPN extending from the right kidney to the scrotum retroperitoneally in a 47-year-old male with uncontrolled blood sugar. The patient was managed with a right percutaneous perinephric drain with right double J (DJ) stenting. His blood sugar was controlled by subcutaneous insulin. The patient was discharged on day 7 in satisfactory general condition with right percutaneous drainage and right DJ stent in situ.

## Introduction

Emphysematous pyelonephritis (EPN) is a suppurative necrotizing form of renal infection with abscess and gas formation in the renal parenchyma and perirenal tissue [1]. It is a rare life-threatening condition and usually presents with flank pain, fever, nausea, and vomiting. If not recognized and treated promptly, the clinical course can be severe and life-threatening. The most common causative organism is *Escherichia coli*, which accounts for 60% of all the cases [2]. The lesion can occur either spontaneously or may associated with a history of trauma and is usually associated with diabetes mellitus in almost all cases [3]. Prompt control of blood sugar and intravenous antibiotics are essential steps in management. Here, we report a case of a newly diagnosed diabetic patient who presented with extensive pyelonephritis extending into the scrotum.

## Case presentation

**Figure 1:**
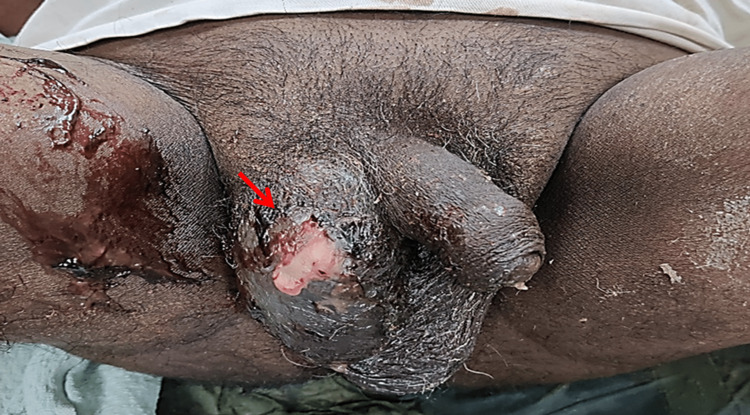
Gangrenous changes with pus discharge (arrow) from right scrotum.

A 47-year-old male was newly diagnosed as diabetic and presented to the clinic with complaints of fever, right flank pain, and pus discharge from the scrotal wall for the last 20 days. On clinical examination, the patient had tachycardia, tachypnoea, and necrotic patch with pus discharge over the right side of the scrotal wall (Figure 1).

**Figure 2:**
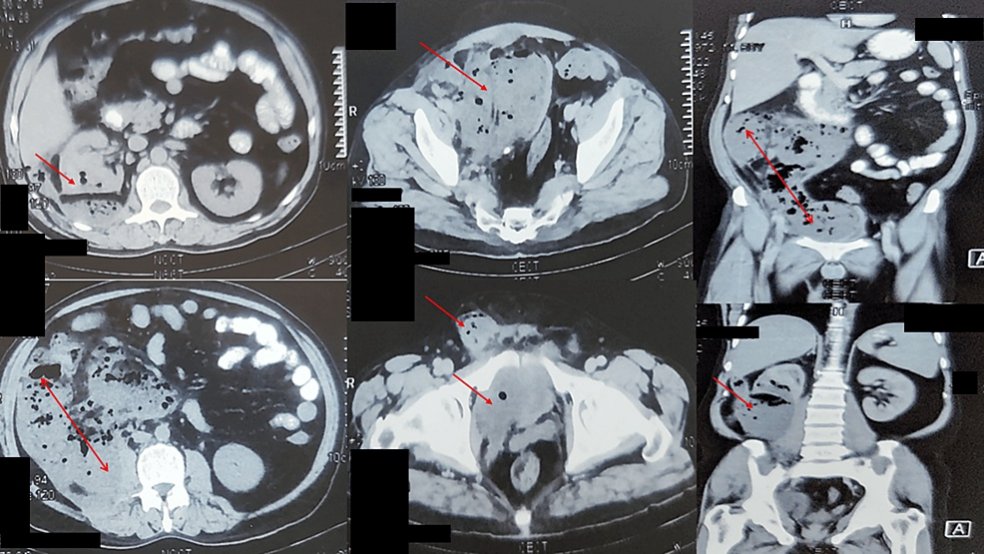
Pre-procedure CECT KUB showing extensive right emphysematous pyelonephritis extended retroperitoneally up to scrotum. CECT: contrast-enhanced computerized tomography; KUB: kidneys ureter bladder

On evaluation, his hemoglobin was 11.7 gm%, total leukocyte count 15,900 cells/mm3, serum creatinine 4.2 mg/dL, blood sugar 221 mg/dL, and glycated hemoglobin (HbA1c) 8.6%. Contrast-enhanced computerized tomography (CECT) abdomen and pelvis revealed a large retroperitoneal collection on the right side extending into the posterior perirenal space and abutting the caecal wall. It showed multiple air pockets (22x15x13 cm) with extension into the right renal capsule, cortex, and right lateral pelvic wall displacing the urinary bladder towards the left. Anteriorly, it extended into the right inguinal region and down to the scrotum, suggestive of right EPN (Figure 2) with normal functioning left kidney. Urine culture showed *Klebsiella pneumoniae*.

The patient was initially managed with intravenous broad-spectrum antibiotics and intravenous fluids. Blood sugar was controlled with subcutaneous insulin. Right-side DJ stenting (5Fr/26) and right-side percutaneous drainage was done under local anesthesia. Incision and drainage of the scrotal abscess were also done at the same time. Debridement was performed and yielded pus only which was sent for culture and sensitivity.

**Figure 3:**
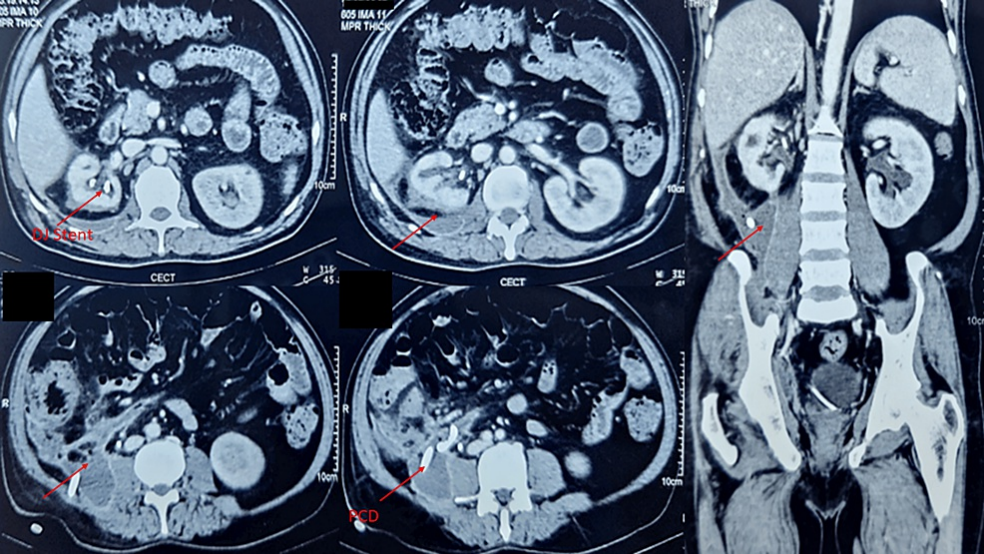
Post-procedure CECT KUB revealed mild irregular walled off collection with embedded air foci in retroperitoneum in right lumber and iliac fossa region with right PCD and right DJ stent in situ CECT: contrast-enhanced computerized tomography; KUB: kidneys ureter bladder; PCD: percutaneous drainage; DJ: double J

Post-procedure CECT KUB revealed mild irregular walled-off collection (180 ml) with embedded air foci in the retroperitoneum in right lumber and iliac fossa region with extension into the pelvis along its lateral wall with normally enhancing bilateral kidneys with right double J (DJ) stent and right percutaneous drainage (PCD) tube in situ (Figure 3).

The patient was discharged on day 7 with stable general condition with right PCD and right DJ stent in situ. On the third week of follow-up, PCD was removed and on the fourth week of follow-up, the right DJ stent was removed. After one month, the patient was able to return to work as a farmer.

## Discussion

EPN is an acute, life-threatening, necrotizing infection of the parenchyma of the kidney and the renal collecting system as well as the surrounding tissues. The hallmark feature of the disease is the presence of gas within these structures. Common risk factors for EPN include diabetes mellitus, obstructive uropathy, and immunosuppression [4]. It is a rare life-threatening condition associated with high mortality, if not treated promptly. Up to 95% of the cases with EPN have underlying uncontrolled diabetes mellitus [5]. The presence of gas in the renal parenchyma is confirmed by ultrasonography or CT and thus supports the diagnosis of EPN. In some cases, gas may extend to the perinephric and pararenal spaces as well as into the scrotum and spermatic cord. Patients are successfully treated by medical management with PCD. This has led to a significant reduction in mortality rates. The treatment protocol may include medical management alone, PCD plus medical management, medical management plus emergency nephrectomy, and PCD plus medical management plus emergency nephrectomy [5].

Our patient presented with right flank tenderness, pus discharge from the scrotal wall, raised serum creatinine, and uncontrolled blood sugar. The infection mostly spread from the kidney to the perinephric space and Gerota’s fascia and then through the flank muscles to the inguinal canal and dartos fascia in the scrotum. However, the pleural and peritoneal cavities were spared and did not show any evidence of infection.

EPN is almost always associated with diabetes mellitus and *E. coli *is the most common causative organism. The other differentials of EPN include acute pyelonephritis with Fournier’s gangrene and xanthogranulomatous pyelonephritis with Fournier’s gangrene.

Several factors are included in the pathogenesis of EPN such as the enhanced proliferation of microorganisms due to decreased immune defenses, fermentation of glucose leading to gas production, and reduced elimination of the gas because of impaired tissue perfusion. Aggressive management including hemodynamic stabilization, intravenous antimicrobial therapy, and diabetes control with insulin therapy is mandatory, but a surgical procedure (nephrectomy or drainage) is almost always required [6]. Mortality remains high despite aggressive treatment in advanced disease.

## Conclusions

This report introduces a rare case of EPN complicated by scrotal extension resulting in gangrenous changes with pus discharge from the scrotum. The triggering factors can be; uncontrolled diabetes, urinary tract obstruction, and hypertension. Aggressive management including hemodynamic stabilization, antimicrobial therapy, control of diabetes, and a surgical procedure (nephrectomy or drainage) is almost always required for adequate patient care and management. We also suggest increased patient surveillance via radiological imaging, to minimize the rates of this pathologic transformation. 
